# Vitamin D and Restless Legs Syndrome: A Review of Current Literature

**DOI:** 10.5334/tohm.741

**Published:** 2023-04-06

**Authors:** Katie L. J. Cederberg, Rosalia Silvestri, Arthur S. Walters

**Affiliations:** 1 Department of Psychiatry & Behavioral Sciences, Stanford University, 3165 Porter Drive Palo Alto, CA, USA; 2 Department of Clinical and Experimental Medicine, Sleep Medicine Center, University of Messina, Azienda Ospedaliera Universitaria “Gaetano Martino”, Messina, Italy; 3 Department of Neurology, Vanderbilt University, Nashville, TN, USA

**Keywords:** restless legs syndrome, vitamin D, genetics, proteomics, cardiovascular disease, inflammation, augmentation, dopamine, iron, endogenous opiate system, serotonin, adenosine, glutamate, vitamin D binding protein, cerebrovascular disease, calcium, phosphorus, parathyroid hormone

## Abstract

This review presents a detailed summary of the current literature regarding RLS and vitamin D deficiency. To our knowledge it is the first review of its kind. We review the prevalence of vitamin D deficiency in RLS as well as the evidence for the use of vitamin D supplementation in RLS management. We further examine the literature for proteomic and genetic evidence of a role for vitamin D in the pathogenesis of RLS. An alteration in vitamin D binding protein in RLS is one of the most consistent findings in the proteomic studies. Furthermore, we examine the interaction of vitamin D with calcium, phosphorus, and parathyroid hormone and the possible role of these connections in RLS. We also explore the possible nexus between RLS and vitamin D in renal disease, cardiovascular and cerebrovascular disease as well as inflammation. In addition, we review the potential interaction between vitamin D and RLS with iron, dopamine and other neurotransmitter systems including the endogenous opiate, serotoninergic, glutamatergic and adenosinergic systems. We also explore the role of vitamin D in RLS Augmentation (i.e., the paradoxical worsening of RLS symptoms when dopaminergic agents are used as a therapy for RLS). Although the literature is not entirely consistent in affirming vitamin D deficiency in RLS or the amelioration of RLS symptoms with vitamin D therapy, the collective studies overall indicate that vitamin D deficiency is common enough in RLS patients to suggest that RLS patients should have their vitamin D levels checked and any deficiency corrected as a standard of care.

**Highlights**

Patients with Restless Legs Syndrome (RLS) may be deficient in vitamin D and therapy with vitamin D may ameliorate RLS. We present the first review dedicated solely to evaluating the relationship between RLS and vitamin D and present a case for the role of vitamin D in RLS pathogenesis.

## Introduction

Restless legs syndrome (RLS), or Willis-Ekbom Disease, is a sensorimotor neurological disorder characterized by the uncontrollable urge to move the legs, in response to uncomfortable or unpleasant sensations, that worsen during periods of rest or inactivity and later in the day or at night. There is at least partial and temporary relief by activity such as walking [1]. The prevalence of RLS is estimated between 5% and 8.8% in the general population of adults and risk factors for developing RLS include sex, age, race, and pregnancy [2]. There is significant health, economic, and societal burden associated with RLS [3,4,5]. Importantly, the pathophysiology of RLS is not well understood; however central iron deficiency and dopaminergic dysfunction are the two leading proposed physiological mechanism(s) that underlie RLS symptoms [6].

The importance of this review is that there is a high prevalence of vitamin D deficiency in people with RLS and there is recent interest in the role of vitamin D in RLS with the use of vitamin D supplementation for managing symptoms. Vitamin D is shown to be deficient in the serum of people with RLS and RLS seems to respond to vitamin D supplementation. Further, vitamin D is related to other well-known pathologies of RLS, including iron deficiency [7,8] and dopamine dysfunction [9,10,11,12]. Serum vitamin D levels can also act as an independent predictor of poor sleep in people with RLS [13]. However, despite the current evidence for a link between RLS and vitamin D, the role of vitamin D in the pathology of RLS is not well understood.

This review presents a detailed summary of the current literature formulating a vitamin D pathogenesis for RLS. We summarize the literature in regard to: (a) the prevalence of vitamin D deficiency in RLS; (b) evidence for the use of vitamin D supplementation in RLS management; (c) proteomic evidence for a vitamin D interaction in RLS; (d) genetic evidence for a vitamin D interaction in RLS; (e) vitamin D interactions with calcium, phosphorus, parathyroid hormone and their possible role in RLS. We also explore the possible nexus between RLS and vitamin D vis a vis (f) the cardiovascular disease and (g) inflammation. Additionally, (h) the possible role of iron, dopamine and other neurotransmitter systems such as the endogenous opiate, serotonerigic, glutaminergic, and adenosinergic systems is explored as playing a role in an RLS and vitamin D interaction. Finally, we examine (i) the possible role of vitamin D in ameliorating the paradoxical worsening of RLS symptoms by dopaminergic therapy (Augmentation). To our knowledge this is the first review of the role of vitamin D in RLS.

## Methods

The literature search was conducted for all published articles prior to and up through February 2023 using the PubMed database. Search terms included “restless legs syndrome and vitamin D”, “restless legs syndrome and treatment”, and “restless legs syndrome and pathogenesis.” Reference lists of included articles were further searched for identification of relevant articles meeting the inclusion criteria not located in the database search. Articles were included in the analysis that: (a) included persons with RLS; (b) included outcomes related to vitamin D; (c) were reported in the English language; and (d) were full-text accessible. Abstracts, book chapters, presentations, and protocol papers were excluded. The search resulted in 94 articles that were pertinent to both RLS and vitamin D.

## Results

### 1. Prevalence of Vitamin D Deficiency in RLS

The summary of 13 studies that presented data related to the rate of vitamin D deficiency and/or RLS is presented in Table 1.

**Table 1:** Summary of studies examining vitamin D levels and restless legs syndrome.


VITAMIN D LEVELS IN PEOPLE WITH RLS										

AUTHOR YEAR	STUDY TYPE	POPULATION	CRITERIA FOR VITAMIN D DEFICIENCY	GROUPS	DIAGNOSTIC/ELIGIBILITY CRITERIA FOR RLS	RLS DIAGNOSIS METHOD	SAMPLE SIZE	N (%) VITAMIN D DEFICIENT	VITAMIN D LEVELS (NG/ML)	NOTES

Wali 2018 [8]	Case-Control	Healthy Adults	<50 nmol/L (<20 ng/mL)	All RLS	2014 IRLSSG [116]	Study-specific questionnaire for diagnostic criteria and associated mimics [116] along with neurological examination of lower limbs	78	59 (75.6%)	12.7 ± 7.0	Secondary analysis controlled for comorbid/clinical conditions
	
				Primary RLS			50	37 (74.0%)	Not reported	
	
				Secondary RLS			28	6 (21.4%)	Not reported	
	
				Controls	Age- and sex-matched at frequency of 2:1 control:RLS ratio		123	52 (42.3%)	26.1 ± 9.9	

Jiménez-Jiménez 2021 [16]	Case-Control	Healthy Adults	N/A	RLS	2014 IRLSSG [116]	Clinical interview in most participants; excluded secondary causes of RLS; excluded conditions: liver, kidney, thyroid and parathyroid diseases, and obesity	285	N/A	21.9 ± 9.7	Excluded participants with known vitamin D deficiency
	
				Controls	Age- and sex-matched		325	N/A	18.6 ± 9.8	

Liu 2021 [15]	Case-Control	Healthy Adults	<50 nmol/L (<20 ng/mL)	All RLS	2014 IRLSSG [116]	Physician interview; excluded secondary causes of RLS, other sleep disorders, diseases that affect vitamin D levels, people taking medications that affect vitamin D levels or drugs that alleviate RLS, and pregnant/lactating women	57	46 (81%)	16.1 ± 5.4	
	
				Mild-Moderate RLS			36	Not reported	17.3 ± 5.4	
	
				Severe RLS			21	Not reported	14.0 ± 4.9	
	
				Controls	Age- and sex-matched		57	1 (2%)	27.0 ± 5.0	

Balaban 2012 [17]	Case-Control	Healthy Adults	N/A	RLS Females	1995 IRLSSG [117]	Clinical interview; no comorbidities and normal neurological examination; excluded known causes of secondary RLS, a familial history of RLS, or any medical conditions that would affect the assessment of RLS	28	Not reported	7.3 ± 4.6	
	
				RLS Males			8	Not reported	11.4 ± 6.2	
	
				Control Females	Age- and sex-matched		27	Not reported	12.3 ± 5.3	
	
				Control Males			11	Not reported	13.0 ± 5.4	

Almeneessier 2020a [19]	Case-Control	Pregnant Women	Normal: Insufficient:	Pregnant RLS	2014 IRLSSG [116]	Clinical interview by trained medical students; excluded conditions that could mimic RLS	223	47 (21.0%)	Not reported	

				Pregnant Control	N/A	N/A	519	66 (12.7%)	Not reported	

Miyazaki 2023 [20]	Case-Control	Pregnant Women	<10 ng/mL and <20 ng/mL	Pregnant RLS	ICSD 3^rd^ [118]	Clinical Phone Interview with Japanese version of CH-RLSq13 [119]	35	LC-MS/MS <10: 12 (34.3%) LC-MS/MS <20: 33 (94.3%)	LC-MS/MS: 11.4 (7.0)	Serum 25(OH)D levels reported as median (IQR) Two different methods of quantifying levels: LC-MS/MS and CLEIA
								CLEIA <10: 24 (68.6%) CLEIA <20: 25 (100%)	CLEIA: 7.2 (6.1)	

				Pregnant Controls	N/A	N/A	168	LC-MS/MS <10: 24 (14.3%) LC-MS/MS <20: 129 (76.8%)	LC-MS/MS: 15.4 (8.1)	
								CLEIA <10: 25 (50.6%) CLEIA <20: 158 (94.0%)	CLEIA: 9.8 (6.3)	

Almeneessier 2020b [21]	Case-Control	Non-pregnant Women	< 25 nmol/L	Non-pregnant RLS	2014 IRLSSG [116]	Clinical interview by trained professionals; excluded comorbid conditions that could mimic RLS and other sleep disorders	271	173 (63.8%)	Not reported	

				Non-pregnant Control	N/A	N/A	865	390 (45.1%)	Not reported	

Bener 2019 [22]	Case-Control	Type II Diabetes Mellitus (T2DM)	Deficient: <20 ng/mL Insufficient: 20-29 ng/mL Sufficient: >30 ng/mL	T2DM RLS	Not reported	Not reported	199	Deficient: 122 (61.3%) Insufficient: 43 (21.6%) Sufficient: 34 (17.1%)	7.7 ± 3.6	

				T2DM No RLS	N/A	N/A	672	Deficient: 289 (43.0%) Insufficient: 214 (31.8%) Sufficient: 169 (25.1%)	8.7 ± 3.8	

Evans 2018 [25]	Case-Control	Healthy Pediatric (3-12 years)	Deficient: <10 ng/mL Insufficient: 11-30 ng/mL Normal: 31-75 ng/mL	RLS	“Yes” to relief from movement	Leg pain questionnaire [120]	12	Deficient: 5 (41.7%) Insufficient: 4 (33.3%) Normal: 3 (25.0%)	15.3 (5.3–61.8)	Vitamin D levels presented as median (minimum-maximum)
	
				GP	“Yes” to questions 1-5		28	Deficient: 4 (14.3%) Insufficient: 20 (71.4%) Normal: 4 (14.3%)	19.7 (4.2–59.3)	
	
				GP+RLS	“Yes” to questions 1-5 and 9		37	Deficient: 10 (27.0%) Insufficient: 24 (64.9%) Normal: 3 (8.1%)	12.8 (4.5–60.7)	
	
				Controls	Age- and gender-matched		13	Deficient: 4 (30.8%) Insufficient: 7 (53.8%) Normal: 2 (15.4%)	15.6 (7.9–61.8)	

Işıkay 2018 [27]	Case-Control	Pediatrics (11–18 years) with Celiac Disease	N/A	Celiac RLS	IRLSSG criteria (version not specified)	Questionnaire – questions/methods otherwise not specified	8	N/A	9.9 ± 4.7	“No patient previously diagnosed with RLS was included in or excluded from the study” RLS severity was negatively associated with serum vitamin D levels

				Celiac no RLS			218	N/A	12.5 ± 11.7	

**RLS IN PEOPLE WITH VITAMIN D DEFICIENCY**										

**AUTHOR YEAR**	**STUDY TYPE**	**POPULATION**	**VITAMIN D STATUS**	**GROUPS**	**DIAGNOSTIC/ELIGIBILITY CRITERIA FOR RLS**	**RLS DIAGNOSIS METHOD**	**SAMPLE SIZE (N)**	**N (%) RLS**	**VITAMIN D LEVELS (NG/ML)**	**NOTES**

Çakır 2015 [28]	Case-Control	Healthy Adults	<20 ng/mL	VDD	2003 IRLSSG 4 criteria [29]	Survey (method not specified); excluded diabetes, vitamin B12 deficiency, chronic renal failure, anemia, and use of any medications that could mimic RLS	57	30 (52.6%)	N/A	
	
			>20 ng/mL	Controls			45	17 (37.7%)	N/A	

Oran 2014 [10]	Case-Control	Healthy Adults	<20 ng/mL	VDD	2003 IRLSSG 4 criteria [29]	Neurologist Evaluation; excluded abnormal levels of ferritin or with a known condition to cause secondary RLS and people with a family history of RLS (among others)	119	60 (50.4%)	11.2 ± 4.7	
	
			>20 ng/mL	Controls			36	6 (16.7%)	34.2 ± 10.0	

Olama 2013 [23]	Case-Control	Premenopausal women with primary fibromyalgia syndrome (PFMS)	≤20 ng/mL	PFMS VDD	2003 IRLSSG 4 criteria [29]	Clinical evaluation; Jenkins’ Sleep Questionnaire [121]; excluded inflammatory rheumatic disease, known osteoporosis, treated with antiresorptive drugs, renal disease, hepatic disease, malabsorption disorder, anticonvulsant therapy, malignancy and pregnancy	28	16 (57.1%)	Not reported	
	
			>20 ng/mL	PFMS Controls			22	6 (27.3%)	Not reported	


*Note*: RLS restless legs syndrome; IRLSSG International Restless Legs Syndrome Study Group; N/A not applicable; ICSD International Classification of Sleep Disorders; CH-RLSq13 Cambridge-Hopkins Restless Legs Syndrome Short Form Diagnostic Questionnaire; LC-MS/MS liquid chromatography–tandem mass spectrometry; CLEIA chemiluminescent enzyme immunoassay; IQR interquartile range; T2DM type II diabetes mellitus; GP growing pains; VDD vitamin D deficiency; PFMS primary fibromyalgia syndrome.

#### 1.1 Prevalence of Vitamin D Deficiency in Otherwise Healthy Adults with RLS

There is substantial evidence for a higher prevalence of vitamin D deficiency in adults with RLS. Within the general population of adults with RLS, a recent meta-analysis including 12 studies related to RLS demonstrated a significant association between serum 25(OH)D levels and the presence of RLS, whereby serum levels of 25(OH)D were significantly lower in 593 people with RLS compared with 1588 controls without RLS [14]. However, that meta-analysis only included four studies of the general population (i.e., otherwise healthy adults) and nine studies in clinical populations (e.g., end-stage renal disease, multiple sclerosis). Two studies demonstrated a higher prevalence of vitamin D deficiency with significantly lower levels of serum vitamin D in people with RLS compared with controls [8,15]. Contradictorily, another study demonstrated significantly higher levels of 25(OH)D levels in idiopathic RLS compared with controls [16]. However, that study excluded participants with a known diagnosis of vitamin D deficiency. Collectively, most studies to date support the premise that the prevalence of vitamin D deficiency is significantly higher in people with RLS.

#### 1.2 Primary vs. Secondary RLS

We identified one study that examined the prevalence of vitamin D deficiency among adults with RLS, whereby 64% of cases had primary RLS and 36% had secondary RLS [8]. That study demonstrated a significant difference in the prevalence of vitamin D deficiency between primary and secondary RLS (OR = 10.4; 95% CI = 1.1–37.5; *p* < 0.001), whereby participants with primary RLS had a 74% prevalence of vitamin D deficiency compared with 21% in secondary RLS [8]. These results suggest that there may be a pathobiological link between vitamin D levels and RLS that is more prominent in the case of idiopathic, or primary, RLS.

#### 1.3 Gender Differences and Pregnancy

There is accumulating evidence for gender differences in vitamin D deficiency within patients who have RLS. One study demonstrated that female adults with RLS had significantly lower levels of serum 25(OH)D compared with females without RLS in addition to significantly lower levels of ferritin, calcium, phosphate, and alkaline phosphatase [17]. However, that study demonstrated no significant difference in serum vitamin D levels between men with RLS and men without RLS [17]. This suggests that vitamin D deficiency is associated with RLS in women, but not necessarily in men.

Regarding pregnancy, a review of the role of vitamin D during pregnancy reported the prevalence of vitamin D deficiency ranges from 10-34% in pregnant women [18]. One study demonstrated a similar prevalence of RLS in pregnant women (30%) compared with non-pregnant women (27%) [19]. However, pregnant women with RLS had a significantly higher prevalence of vitamin D deficiency (21%) than pregnant women without RLS (13%), and vitamin D deficiency was a significant and independent predictor of RLS in pregnant women (OR 2.376 [CI 1.488–3.794], *p* < 0.001) [19]. A recent study examined the optimal cutoff value of 25(OH)D for determining RLS in pregnant women using two different methods of measurement [20]. Vitamin D levels were significantly lower in pregnant women with RLS than pregnant women without RLS (i.e., controls) for both methods and there was a significantly higher proportion of pregnant women with vitamin D deficiency with RLS compared with controls using LC/MS, but no difference in classification of vitamin D deficient using CLEIA. They further determined an optimal LC-MS/MS cutoff value of 10.0–12.7 ng/mL for classifying RLS in pregnant women in their third trimester, while the estimated cutoff value for nonpregnant women of the same age was 14.8–18.8 ng/mL [20]. These results suggest that the prevalence of vitamin D deficiency is higher during pregnancy. However, the higher prevalence of vitamin D deficiency in women may not be specifically associated with pathobiological changes during pregnancy.

Another study in non-pregnant women of childbearing age demonstrated that 24% of non-pregnant women had RLS and 76% did not have RLS [21]. That study further demonstrated a higher prevalence of vitamin D deficiency in women who had RLS (64%) compared with women who did not have RLS (45%) and that vitamin D deficiency was a significant and independent predictor of RLS in non-pregnant women, along with diabetes [21]. Although prevalence estimates were higher, these results are consistent with the study of non-pregnant women and suggest that vitamin D deficiency is significantly higher in women with RLS compared with controls without RLS regardless of pregnancy status.

#### 1.4 Adults with other comorbid/clinical conditions

Adults with RLS who have other clinical or comorbid conditions may have a greater association and prevalence of RLS and vitamin D deficiency. A subgroup analysis in the aforementioned meta-analysis including 12 studies related to RLS and vitamin D deficiency demonstrated a significant association in people with end-stage renal disease and those with other comorbidities (e.g., multiple sclerosis, celiac disease, ankylosing spondylitis, dialysis, migraine, lung transplant) compared with people who only presented with RLS [14]. Another study demonstrated a significant and independent association between vitamin D deficiency and the presence of RLS after adjusting for all other significant clinical factors (odds ratio = 3.1 [95% confidence interval = 1.51–6.38]; *p* < 0.002) [8]. It is important to note that study only included skin color, education, occupation, monthly income, and marital status as clinical factors in multivariate analyses, and not comorbid or clinical conditions that are highly associated with RLS and vitamin D deficiency. Another study in people with type II diabetes demonstrated that 22.8% of participants had RLS and within the group who had RLS and type II diabetes, 61.3% had vitamin D deficiency and 21.6% had vitamin D insufficiency [22]. That study confirmed the high prevalence of RLS in adults with type II diabetes and further suggests an integral role of vitamin D.

There have been similar findings in conditions related to musculoskeletal pain including fibromyalgia. One study found that women with primary fibromyalgia syndrome (PFMS) who were vitamin D deficient were more likely to have RLS than those with PFMS with normal vitamin D levels [23]. Collectively, these findings suggest that the prevalence of vitamin D deficiency in RLS may be related to other clinical factors, and it is necessary to consider the presence of comorbid conditions in the assessment and treatment of vitamin D deficiency and RLS.

#### 1.5 Pediatric Populations

Although pediatric RLS is less understood, there is recent evidence for an association with vitamin D in children and adolescence [24]. One study examined the correlates and predictors of leg pains. They divided the group into those with growing pains (n = 28), RLS (n = 12), and a mixed group (n = 37) that had both growing pains and RLS. They found that 41.7% of children with RLS were vitamin D deficient compared with 14.3% of those with growing pains and 30.8% of controls. Further, 8.3% of RLS patients were anemic compared with 10.7% of those with growing pains and 0% of controls [25]. This suggests that the presence of RLS in children may be related to vitamin D deficiency and anemia. Of note, although RLS and growing pains are different conditions, there are significant overlaps in symptom presentation and vitamin D abnormalities are reportedly associated with both RLS and growing pains which may complicate differential diagnosis [26].

Another study demonstrated a similar prevalence of RLS in children with celiac disease (CD) compared with controls (3.5% and 3.0%, respectively); however, patients with CD and RLS had significantly lower vitamin D and ferritin levels compared with patients with CD who did not have RLS [27]. Additionally, increased severity of RLS within the CD group was significantly and negatively correlated with lower serum ferritin, folic acid, and 25(OH)D levels [27]. Results from that study suggest that, although the prevalence of RLS is not necessarily higher in children with CD compared to control children, the presence and severity of RLS within children who have CD may be related to low vitamin D and ferritin levels.

#### 1.6 Prevalence of RLS in Vitamin D Deficiency

There is evidence of an increased prevalence of RLS within adults who are vitamin D deficient. One study demonstrated that 53% of people with vitamin D deficiency had RLS compared with 38% of the group with normal vitamin D levels [28]. Of note, that study found no significant difference between groups for levels of iron, ferritin, magnesium, or hemoglobin [28], suggesting that the prevalence of RLS in adults with vitamin D deficiency may not be related to other clinical factors commonly associated with idiopathic RLS. Further, the prevalence estimates of 53% and 38% are high compared with what is expected from the general population with RLS, which could be a result of the authors utilizing only the four essential diagnostic criteria without the exclusion of mimics [29] and a questionnaire-based diagnosis. Another study demonstrated that the presence of RLS was significantly higher in patients with vitamin D deficiency (50%) compared with patients with normal vitamin D (17%). That study further demonstrated that serum 25(OH)D levels and the classification of vitamin D deficiency were significant predictors for the presence of RLS [10]. However, that study excluded participants with abnormal levels of ferritin or with a known condition to cause secondary RLS and people with a family history of RLS, potentially excluding some adults with primary RLS.

### 2. Vitamin D Levels Associated with RLS Severity

In addition to the association between the presence of RLS and vitamin D, there is further evidence for a relationship between the severity of RLS and vitamin D. Three studies have demonstrated a significant, negative association between RLS severity and 25(OH)D (i.e., higher RLS severity was associated with lower serum vitamin D levels) [15,17,30]. Similarly, a study in 59 people with RLS and 52 controls found that mean RLS severity was higher for patients who had both RLS and vitamin D deficiency than for patients with RLS who had normal vitamin D levels [8]. Two additional studies, one in 29 patients with RLS on hemodialysis and the other in 57 otherwise healthy adults with RLS, demonstrated that vitamin D deficiency was significantly higher in adults with severe-to-very severe RLS compared with mild-to-moderate RLS [15,31]. Although 25(OH) vitamin D levels and vitamin D deficiency did not differ between patients with RLS on hemodialysis and non-RLS controls on hemodialysis. Similar findings have been reported in children with RLS, whereby one study found that RLS severity was significantly and negatively correlated with serum ferritin, folic acid, and 25 (OH) vitamin D levels in children with celiac disease [27]. Collectively, these studies demonstrate a significant association between RLS severity and vitamin D deficiency in adults and children.

### 3. Effect of Vitamin D Supplementation on RLS

There is clear evidence of the relationship among the presence and severity of RLS and vitamin D deficiency; however, the evidence for vitamin D supplementation to manage RLS is mixed. The summary of studies that have examined the effect of vitamin D supplementation on RLS is presented in Table 2. A recent randomized controlled trial comparing vitamin D supplementation (50,000 IU caplets) with a placebo control in 35 people with RLS found no significant change in RLS severity after 12 weeks [32]. Importantly, this study did not include patients with vitamin D deficiency and suggested such an examination in a larger sample who were vitamin D deficient. A case study in 45 year old woman with Turner syndrome and RLS who had 25 (OH) vitamin D deficiency found that vitamin D supplementation returned serum vitamin D levels to normal and improved RLS symptoms [33]. Another study in 12 patients with primary RLS and vitamin D deficiency demonstrated a significant and clinically meaningful reduction in RLS severity (IRLS score of 26 [range = 15–35] to 10 [range = 0–27]) after vitamin D3 supplementation (28,000 IU/week oral or 200,000 IU/month intramuscular with calcium carbonate and maintenance dose of 400 IU/day) that were continued until levels were corrected [34]. However, there were major limitations to that study including the lack of a control group and non-blinding. Correspondingly, another study provided vitamin D supplementation (50,000 units/week) for 2 months to 21 patients with RLS who were vitamin D deficient. A total of 19 (90%) patients had vitamin D levels rise to sufficient levels with supplementation and were included in analyses, which demonstrated a significant and clinically meaningful decrease in RLS symptoms including overall IRLS scores and sub-scores related to symptom severity, impact on sleep, symptom measures, and disease impact measures [35]. Of note, although the change in RLS severity was clinically meaningful, the total IRLS scores changed from 24.9 to 21.1 indicating that the average remained within the severe RLS classification. Furthermore, this study also lacked a control group condition. Collectively, these results suggest that vitamin D supplementation, in those with RLS who are vitamin D deficient, could improve symptom severity and may represent a safe and effective method to managing symptoms. However, more clinical randomized trials with control conditions are necessary to fully understand the efficacy and effectiveness of vitamin D supplementation for managing RLS.

**Table 2:** Summary of studies examining the effect of vitamin D supplementation on symptoms of Restless Legs Syndrome.


FIRST AUTHOR, YEAR (CITATION)	POPULATION	STUDY TYPE	SAMPLE SIZE	DOSAGE	DURATION	CONTROL CONDITION	OUTCOME

Wali, 2019 [32]	RLS with normal vitamin D levels	Randomized Double-blind Placebo-controlled Trial	35 (17 vitamin D group; 18 placebo group) 22 completed study (12 vitamin D; 10 placebo)	50,000 IU orally weekly	12 weeks	Placebo (orally)	No significant change in RLS severity from baseline (IRLS = 14.6) to follow up (IRLS = 14.5) in the vitamin D group (*p* = 0.540)

Buratti, 2017 [33]	Turner syndrome and RLS with vitamin D deficiency	Case Study	1	Not Specified	6 months	N/A	Normal vitamin D levels achieved and complete recovery in RLS symptoms after 6 months

Wali, 2015 [34]	Primary RLS with vitamin D deficiency	Longitudinal Study	12	28,000 IU oral dose/week or 200,000 IU intramuscular injection/month with 400 IU daily maintenance dose	Continued until vitamin D levels corrected (range: 3 to 8 months)	N/A	RLS severity improved from baseline (IRLS = 26) to when the vitamin D levels were corrected (IRLS = 10; *p* = 0.002)

Tutunca, 2019 [35]	Idiopathic RLS with vitamin D deficiency	Longitudinal Study	21 (19 included in analyses)	50,000 IU/week	2 months	N/A	Vitamin D levels increased (13.2 to 42.8 ng/mL) and RLS severity improved (IRLS = 24.9 to IRLS = 21.1; *p* < 0.001).


*Note*: *RLS* restless legs syndrome; *IU* international units; *IRLS* International Restless Legs Syndrome Severity Score.

### 4. Proteomic Evidence of Vitamin D Deficiency in RLS

There is recent interest in utilizing blood-based biomarkers, including proteomics, to ascertain the presence and severity of sleep conditions [36]. To date, four studies have examined proteomic biomarkers of RLS [37,38,39,40]. Three studies identified significant differences in levels of vitamin D binding protein (i.e., Group-specific Component [GC]) between people with RLS and controls; however, the direction of differential expression was inconsistent across studies. The first study was conducted using cerebrospinal fluid (CSF) from 5 people with early-onset RLS compared with 5 age- and sex-matched controls and demonstrated a significant upregulation of vitamin D binding protein (fold change[FC] = +2.3) in patients with RLS [38]. Similarly, another study using serum samples from 7 people with RLS compared with 6 age- and sex-matched controls demonstrated a significant upregulation of vitamin D binding protein (FC = +3.5) in people with RLS [39]. The most recent study using serum samples from 12 people with RLS compared with 10 healthy controls similar in age and sex demonstrated a significant downregulation of vitamin D binding protein (FC = 0.94) in people with RLS [37]. Vitamin D binding protein is associated with the binding of vitamin D and its metabolites and transports them to the target tissue; however, the regulation of vitamin D binding protein is regulated by estrogen, glucocorticoids and inflammatory cytokines and not vitamin D levels directly [41]. It has also been associated with functions related to scavenging for toxins released after cellular injury/death as well as a modulator of immune response. An upregulation or downregulation of this protein might suggest irregular inflammatory and immune responses (discussed later).

One reason for the inconsistency in direction of association may be due to key methodological differences among the studies. All three studies used mass spectrometry techniques; however, one study used two-dimensional difference gel electrophoresis (2D-DIGE) [38], one study used two-dimensional gel electrophoresis (2-DE) [39], and one study used liquid chromatography mass spectrometry (LC-MS) [37]. All studies reported collecting samples in the morning; however, the time frame of collection varied among studies from a small range (e.g., between 7:00 and 8:00) to a larger range (between 8:00 and 12:00). Additionally, one of the serum studies specified collecting fasted blood samples [39], whereas the other did not specify. The two serum studies further described using techniques to remove the most abundant proteins to reduce the high dynamic range, or complexity, of the samples; however, each study removed a different number of abundant proteins ranging from two to 14 removed proteins. Of note, one study reported removing haptoglobin and antitrypsin as abundant proteins and later identified haptoglobin and alpha-1 antitrypsin as significantly downregulated in patients with RLS [37]. Additionally, the studies included relatively small sample sizes (range: 8–17 RLS patients) and quantified different arrays of proteins (range: 272–492 proteins in plasma and 663 proteins in CSF proteins quantified and included in analyses). Presently, the comparison of significant findings is limited among studies conducted in RLS. However, we would point out that in each of these studies that the changes in vitamin D binding protein were discovered with a hypothesis free approach. Thus, the fact that changes in vitamin D binding protein were discovered in 3 of 4 studies makes it highly unlikely that these results are by chance alone. We would also point out that it is not uncommon for CSF results to be the opposite of those found in plasma due to blood brain barrier and other considerations.

### 5. Genetic Evidence of Vitamin D Deficiency in RLS

In a study of 205 patients with RLS and 445 controls, the presence of 2 single nucleotide polymorphisms (VDR rs2228570 and VDR rs731236) in the vitamin D receptor was studied. The distribution of allelic frequencies of the 2 SNPs and the distribution of their genotypes was similar independent of the presence or absence of a family history of RLS. The frequencies of the rs731236AA genotype (P < .005) its allelic variant rs731236A (P < .01) were statistically significantly lower in RLS patients. There was no difference between patients and controls in the distribution of the other SNP VDR rs2228570 or its allelic variants [42].

In another study of 285 RLS patients and 325 controls, the group then repeated the study on VDR rs731236 which had previously been shown to be less associated with RLS, but this time did not show any relationship. They also repeated the aforementioned study on VDR rs2228750 and showed no relationship to RLS as previously discovered. They then looked at other vitamin D receptor genes and also at GC vitamin D binding protein (GC) genes (VDR rs7975232, VDR rs739837, VDR rs78783628, GC rs7041, GC rs4588) in relation to RLS and results were also non-associative. There was also no relationship of any of these SNPs to a family history of RLS [16]. That vitamin D protein binding genes did not show any relationship to RLS in this study is in contrast to proteomic studies, which show that vitamin D binding protein itself is altered in cerebrospinal fluid (CSF) and serum in RLS patients. They then looked at the relationship of these SNPs to serum vitamin D levels and did not find any. Interestingly, however, serum 25-hydroxyvitamin D levels were significantly higher in the RLS patients than the controls (P = .0002) which is contradictory to previous studies where serum vitamin D levels had been shown to be low in RLS. However, a key limitation to this study was the exclusion of participants with a known diagnosis of vitamin D deficiency. Another interesting finding, however, was that RLS patients who had the rs7975232CC genotype or rs7975232C allele had a higher response to GABAergic medications [16].

One of the genes found to be more highly associated with RLS in Genome Wide Association Studies (GWAS) is the Meis-1 gene. In an RNA-sequencing study of cell lines where Meis-1 was either over-expressed (SK-N-SH cells) or under-expressed (HEK293 cells), it was found that mineral absorption pathways were highly involved influenced by Meis-1 and that vitamin D receptor gene was one of those activated in the mineral absorption pathways [43].

### 6. Vitamin D Interaction with Calcium, Phosphorus, Parathyroid Hormone and their Possible Role in RLS

Normally, vitamin D is converted into its active form by two steps, the first in the liver and the second in the kidney. This active form of vitamin D (1,25-dihydroxy vitamin D) then absorbs dietary calcium from the gut to increase serum calcium levels so that calcium can be transported and incorporated into bone for the maintenance of bone growth and sturdiness [44]. Under conditions of renal failure, vitamin D is not able to be as actively converted to its active form by the kidney so that not as much calcium is absorbed from the GI tract resulting in hypocalcemia. Parathyroid hormone then increases to try and stimulate more of the conversion of vitamin D into its active form (secondary hyperparathyroidism). In renal failure hyperphosphatemia also occurs because of inadequate ability of the kidney to excrete phosphate [45].

Restless Legs Syndrome is a common accompaniment of renal failure and virtually all of the studies on calcium metabolism in RLS have occurred in RLS patients with renal failure. A review of 36 studies in RLS patients, most of whom were undergoing dialysis, indicated that phosphorus levels are even higher and vitamin D levels even lower in patients with renal failure and RLS than in renal failure patients without RLS. There was no difference in serum calcium [14]. Another similar meta-analysis of 23 studies, however, found no difference in serum phosphorus, calcium and parathyroid hormone between dialysis patients with or without RLS [46]. On the other hand, two studies each of which included 10 RLS patients with renal failure and secondary hyperparathyroidism found that the RLS severity improved after parathyroidectomy [47,48].

### 7. Pathobiological Links Between Vitamin D and RLS

#### 7.1 Inflammatory and Autoimmune Connections

There is a large and growing body of evidence that vitamin D is involved in preventing inflammation. The active form of vitamin D affects every level of the immune system from macrophages (phagocytosis plus other functions) and dendrites (antigen presenting cells) to B cells (antibody production or humoral immunity) to T cells (cellular immunity). In general, the role of active vitamin D is to dampen the inflammatory and autoimmune responses. For example, the active form of vitamin D inhibits the ongoing proliferation of activated B cells and induces their apoptosis and thus inhibits antibody production in in-vitro studies. In in-vitro studies, the active form of vitamin D also inhibits the conversion of B cells to plasma cells thus further limiting antibody production by the plasma cells [49]. However, attempts to reproduce these results in-vivo, have not been successful, in Multiple Sclerosis, for example [50]. In regard to T cells, the active form of vitamin D suppresses the differentiation of Th1 cells which are pro-inflammatory and stimulates the differentiation of Th2 cells which are anti-inflammatory. The active form of vitamin D also promotes the differentiation of Treg cells which play an important role in preventing auto-immune disease [51].

RLS seems to be more frequently associated with inflammation and auto-immunity than would be expected by chance alone. In a literature review of individually published studies, it was found that forty two of forty-seven (89%) of other medical disorders that are reported to be more frequently associated with RLS, are also disorders that are characterized by inflammatory or immune changes. Examples include Multiple Sclerosis, Rheumatoid Arthritis and Celiac Disease [52]. This suggests that RLS can be triggered by inflammatory of auto-immune mechanisms. Other evidence to support this position was also elicited in the review including evidence that RLS is triggered by infection, RLS is responsive to steroids under blinded conditions and inflammatory markers are elevated in RLS [52]. Other subsequent studies have broadly supported these findings including new reports of inflammatory markers in RLS [53], a report that polymorphisms of the cytokine genes interleukin-1 Beta and Interleukin -17 alpha are more frequently associated with RLS [54] and a most recent publication showing that RLS is more frequently associated with Long COVID in women [55]. Whether vitamin D could alter the infection, inflammation or auto-immunity associated with RLS bears more study.

#### 7.2 Connection to Heart Disease and Stroke

Numerous studies have shown that vitamin D deficiency is linked to a higher prevalence of cardiovascular and cerebrovascular disease [56,57,58,59]. In the case of stroke, the link is more to ischemic rather than hemorrhagic stroke [59]. Proposed mechanisms include adverse effects upon the known roles of vitamin D in the maintenance of endothelial function, its anti-inflammatory and anti-oxidant properties, its role in the renin-angiotensin-aldosterone regulatory pathway and its maintenance of autophagy (i.e., breakdown/destruction of old, damaged, or abnormal cellular components) [56,57,58]. However, studies of vitamin D supplementation have not shown convincing evidence that such supplementation can prevent the onset of cardiovascular or cerebrovascular disease.

Numerous studies have now shown a link between cardiovascular disease, cerebrovascular disease and RLS [60]. Although there are some contradictions, most cross-sectional and prospective epidemiology studies support such as association. In addition, in-lab studies have shown the connection between cardiac arrythmias such as atrial fibrillation and non-sustained ventricular tachycardia and periodic limb movements during sleep (PLMS) [61,62] and between echocardiographic abnormalities such as left ventricular hypertrophy and RLS/PLMS [63,64,65]. Hypoxic markers are also increased in RLS compared to controls [53,66,67,68]. The changes in two protein biomarkers, originally discovered through proteomic studies, were verified by enzyme-linked immunosorbent assay (ELISA) in plasma samples from RLS patients and controls and kininogen-1 (KNG 1) was significantly higher and alpha-1- antitrypsin (A1AT) significantly lower in RLS patients than controls. As is previously known from the literature, high levels of KNG1 and low amounts of A1AT are related to an increased risk for cardiovascular disease [69]. Recent studies have also shown increased silent cerebral microvascular disease, a pre-stroke condition, in RLS patients without a previous history of clinical stroke as demonstrated by both MRI and autopsy studies [70,71]. PLMS are also more highly associated with increased cerebral microvascular disease in patients with first-ever minor stroke or high-risk transient ischemic attack as shown by MRI [72]. A recent epidemiology study suggests that treating RLS with RLS-specific medications lowers the cardiovascular risk associated with RLS [73]. More specific cardiovascular/cerebrovascular RLS oriented studies with a vitamin D specific focus need to be performed to explore these relationships further.

### 7.3 Vitamin D and Dopaminergic Pathways

Three dopaminergic agents have been approved for treatment of RLS by the FDA and European counterparts. In addition, autopsy studies and neuroimaging studies have suggested there is upregulation of dopamine at the level of the striatum [74,75]. Early studies [9] demonstrated that chronic vitamin D deficiency significantly increased dopamine content in the hypothalamus and cortex of the weanling rat compared to rats on a regular diet or vitamin D-replete regimen. Later studies [76] demonstrated a neuroprotective effect of vitamin D on dopaminergic cells exposed to glutamate. In chronic pain patients with RLS, vitamin D3 deficiency correlates with higher pain and lower quality of life [77]. A pathogenetic mechanism involving reduced glutathione content potentially responsible for selective dopaminergic neuron demise has been proposed by Oran and colleagues [10] in RLS patients. Vitamin D deficiency affects the development of dopaminergic neurons in early life, as suggested by an increased vitamin D receptor (VDR) expression in the rat midbrain until weaning, as well as in the human substantia nigra [78].

Vitamin D has been strongly associated with the production of tyrosine hydroxylase, the rate-limiting enzyme for dopamine [79]. Dopamine levels were decreased in vitamin D (DVD)-deficient neonatal rats, later affecting the animal’s adult behavior leading to increased locomotion. The DVD-deficient rats, females more than males, were also selectively sensitive to amphetamine induction of presynaptic dopamine release [11]. Moreover, these rats were particularly sensitive to postsynaptic D2 blockade by haloperidol.

Vitamin D supplementation with 2000 IU/day for 12 weeks was successfully employed in ADHD children to increase both vitamin D and dopamine [80].

#### 7.4 Endogenous Opiates

Opioids are considered one of the mainstays for RLS treatment and are approved in many countries in Western Europe for the treatment of RLS [81]. In autopsy studies of RLS, beta endorphin, and perhaps metenkephalin, are reduced in the thalamus [82]. A mu opiate receptor and total opiate receptor knockout mouse have also been created which mimic many of the motor, sensory, and biochemical features (e.g., iron, dopamine) of RLS [83,84,85].

Vitamin D deficiency may alter muscle sensitivity via hyperinnervation of nociceptors in skeletal muscle tissue leading to pain hypersensitivity [86]. The mechanism of action of D_3_ in pain management supplementation likely relies on its anti-inflammatory effects mediated by reduced cytokine and prostaglandin release. A recent review suggests that VDR may play a role in modulating the expression of pain genes and ion channels in nociceptor neurons [87].

Vitamin D would also directly or indirectly affect nerve growth factor expression from the dorsal root ganglia or hippocampal neurons. In fact, nerve growth factor is critical to pain processing, modulating the transcription of different isoforms of sodium channels and promoting the release of calcitonin gene-related peptide. In addition, the glial cell line-derived neurotrophic factor and the epidermal growth factor receptor, both involved in pain processing, are also regulated by the vitamin D pathway and voltage-gated calcium channels. Allodynia, cerebral dysregulated nociception, shares 21 opioid-associated genes with vitamin D metabolism [88].

#### 7.5. The Serotonin System

Serotonergic reuptake inhibitor antidepressants exacerbate RLS symptoms. In a single photon emission tomography (SPECT) study, RLS severity was found to be increased when there was decreased availability of serotonin transporters in the pons and medulla. These data support the hypothesis that an increase of serotonergic neurotransmission may exacerbate RLS symptoms. [89].

Vitamin D regulates the transcription, synthesis, release, and function of serotonin in the brain. Vitamin D mediates a functionally opposite regulation of tryptophan hydroxylase (TPH) 1 and 2, the rate-limiting step in serotonin synthesis. Dysfunctional serotonin activation and function during critical periods of development could be susceptible to simple vitamin D_3_ supplementation.

In an experimental model, a low vitamin D diet reduces cerebral serotonin in mature female mice and contributes to weight gain in a favorable environment [90]. In a healthy population, vitamin D increase via UVB exposure promotes general well-being through positive mood and sleep modulation [91].

The anti-depressive effect of vitamin D supplementation was reported in a study [92] providing 50,000 IU for three months to adults with major depressive disorder. The intervention significantly improved Beck Depression Inventory scores and serotonin levels compared to a control group. While serum serotonin levels were equally increased in both genders, there was a significant difference in terms of depression score improvement between males and females, favoring the female gender.

#### 7.6 The Glutamate System

In RLS, gabapentin and pregabalin are two of the major treatments for RLS [93,94,95,96]. They are thought to inhibit glutamate release via their effect on the alpha-2-delta calcium channel. In proton Magnetic Resonance Spectroscopy (MRS) studies there is evidence of increased glutamate at the level of the thalamus in RLS patients as determined by the Glx/creatinine ratio where Glx is defined as a combination of glutamate and glutamine, but mostly glutamate [75,97]

Glutamate is the primary excitatory neurotransmitter in the central nervous system. Excessive glutamate release and/or impaired glutamate removal causes prolonged activation of postsynaptic receptors, resulting in excitotoxicity. Vitamin D can attenuate the resulting hyper-intracellular calcium concentration and reduce glutamate excitotoxicity in a dose-dependent fashion. Vitamin D deficiency (VDD) during puberty in rats causes presynaptic malfunctioning via alterations in glutamate uptake by reducing the expression of glutamate (EAAC-1) transporters [98].

In a recent experimental mouse model [99], VDD was associated with intensified reactive oxygen species production and increased presynaptic calcium ion concentrations, whereas one-month VDD correction only partially recovered glutamate dysfunction and the proinflammatory shift.

#### 7.7 The Adenosine System

Restless legs syndrome is responsive to dipyridamole, which increases adenosine by blocking cellular uptake [93,100]. Animal models have indicated that in the presence of iron deficiency, adenosine levels are decreased resulting in enhanced glutamate levels, which can lead to neurotoxicity, as discussed above. Adenosine acts as a sedative depressant agent, promoting sleep and suppressing arousal. Adenosine A1 receptors have been implicated in the hyperarousal state typical of RLS, whereas striatal A2A receptors are related to dopaminergic signaling in brain iron deficiency [12].

Adenosine drives the inflammatory response in damaged tissue by accumulating concentrations during the active phase of inflammation and promotes immune cell functional repolarization to resolve the inflammatory process [101]. Vitamin D also exerts an immune-modulating effect on two cell-surface ectonucleotidases, CD39 and CD73, and adenosine deaminase enzymes, thus regulating adenosine levels [102]. Vitamin D supplementation reduces arthritis inflammation [103] and protects from autoimmune illnesses by lowering the activated cell response in the context of the inflammatory process [104].

#### 7.8 Vitamin D, Anemia, and Iron

In RLS, oral and intravenous iron supplementation are a mainstay of treatment. Both peripheral and central iron deficiency are implicated in RLS via serum, CSF, postmortem, and neuroimaging studies [75,93,105,106].

Vitamin D is a potent regulator of the hepcidin-ferroportin axis in humans. A single oral dose (100,000 IU) was shown to decrease the circulating levels of hepcidin by 34% within 24 hours of supplementation [107]. Vitamin D has been previously associated with anemia in various healthy and diseased populations. However, this association differs between races and ethnic groups and is likely specific to anemia of inflammation [7]. Vitamin D, in fact, suppresses hepcidin mRNA transcription and supports erythropoiesis and iron homeostasis. There is significant VDD and iron-deficient anemia in RLS patients compared to a control population [8].

Vitamin D supplementation reduces proinflammatory cytokines and erythropoiesis-stimulating agent requirements in patients with chronic kidney disease (CKD), mobilizing iron stores. Besides CKD, combined IV iron and vitamin D supplementation should be considered in RLS with celiac disease. In the latter, in fact, RLS severity is negatively correlated with serum ferritin, folic acid, and 25(OH) vitamin D levels [27].

Vitamin D deficiency and a 6.5-fold higher RLS prevalence than idiopathic RLS are observed in uremic patients with CKD [108]. The accumulation of uremic toxins results in neurotoxicity with damage to the blood-brain barrier, oxidative stress, apoptosis, and metabolic acidosis with hyperphosphatemia preventing the activation of vitamin D, resulting in hypocalcemia.

### 8. The Role of Vitamin D in Augmentation

Diker [109] reported significant clinical improvement of dopaminergic augmentation experienced by an 81-year-old female by slow tapering off of offending agents, cotreatment of iron-deficient anemia, and vitamin D supplementation. Symptoms were fully resolved after eight weeks of D_3_ supplementation (50,000 IU/week). Symptom-free maintenance was achieved by replacing pramipexole with gabapentin first, then pregabalin (150 mg/day), iron capsules, and 400 IU/day of oral vitamin D.

Preliminary unpublished results from a small cohort of augmented patients in our Sicilian Sleep Center showed a prevalent female VDD in 9 RLS patients (5 F) on dopaminergic drugs and a consequent female improved response to vitamin D supplementation. Augmentation in our cohort was also related to decreased ferritin levels, OSA, and length of DOPA-agonist therapy; in females, other significant factors were vitamin D levels and postmenopausal age. Augmentation was resolved by progressively tapering off dopamine agonists in favor of pregabalin and co-treating with iron and vitamin D supplementation as needed.

## Discussion

To our knowledge this is the first review, to our knowledge, dedicated solely to evaluating the relationship between RLS and vitamin D that also presents a case for the role of vitamin D in the pathogenesis of RLS and a rationale for a possible add-on treatment. The merit of this review is not only to report on the prevalence of vitamin D deficiency in RLS and RLS response to vitamin D supplementation, but to further characterize populations at risk for RLS within vitamin D deficiency and to predict vitamin D response among different phenotypes of RLS.

So far, we have learned that vitamin D deficiency is more related to idiopathic rather than secondary RLS [8], especially in women who have a prominent risk compared to males during specific times of their reproductive life (i.e., pregnancy and menopause). Of note, however, vitamin D deficiency would also predict the occurrence and severity of symptoms in adults with RLS comorbid clinical conditions such as, in particular, ESRD [14], diabetes [22], PFMS [31] and children with Celiac disease [27] or ADHD [80]. As for the pediatric population with leg pain, vitamin D deficiency is found three times more often in RLS than in growing pains [25], whereas iron deficiency anemia, also common to both RLS and growing pains, does not separate the two populations in terms of prevalence.

Also, increased severity of symptoms in RLS vitamin D deficient patients, both in primary and secondary forms, correlates with vitamin D levels; an important finding to foster vitamin D supplementation among RLS therapeutic tools. This therapeutic effect could be related to different vitamin D mechanisms including the known effect on mood [110], pain [87,88] and sleep. However, the variability of methods, clinical and control populations, vitamin D dosing and length of follow-up, would require more randomized trials with controlled conditions to fully understand and characterize patients most susceptible to vitamin D replacement.

**Figure 1:**
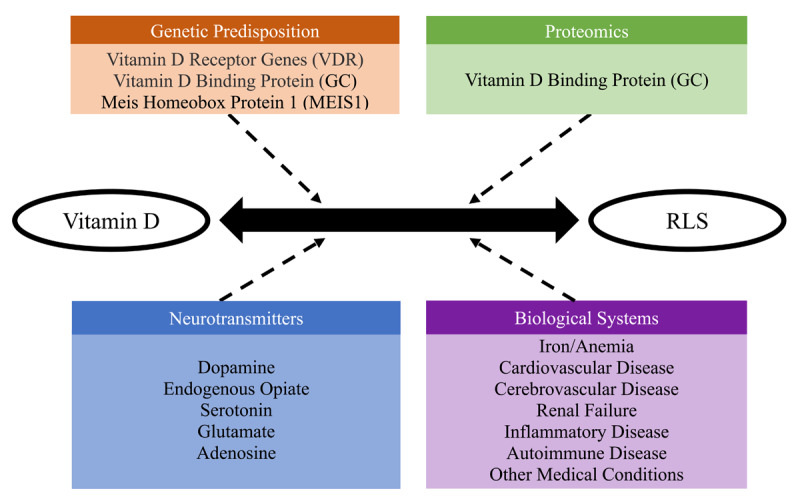
Hypothetical influences on the interaction between vitamin D and restless legs syndrome.

Genetic predisposition, proteomics, neurotransmitter and biological system mechanisms are likely to link vitamin D to RLS (see Figure 1). Since much of the research summarized in this review was done on populations not expected to be Vitamin D deficient, it would seem that the vitamin D deficiency in RLS is not merely a matter of dietary lack. From the evidence accumulated so far, it would seem that there are intrinsic mechanisms specific to RLS itself that account for the vitamin D deficiency. More genetic and biochemical research needs to be done. A key question is whether RLS patients are vitamin D deficient because of decreased synthesis or increased breakdown of vitamin D. SNPs for enzymes that are involved in the formation of vitamin D and its breakdown could be investigated and, for these same enzymes, levels in blood and CSF could be investigated in RLS patients and controls.

Despite some conflicting evidence from proteomic and genetic studies, an alteration of vitamin D binding protein is consistently reported by three out of four proteomic studies [37,38,39,40]. This is highly significant since each of these results were found in hypothesis free experiments where literally hundreds of other proteins were also investigated. The proteomic studies should be repeated with RLS patients and controls getting both serum and CSF samples at the same time in conjunction with serum vitamin D levels.

The well-known effects of vitamin D on inflammation, autoimmune response, and its protective role against cardio and cerebrovascular disease as well as amyloid deposition and glutamate neurotoxicity, may also explain some dire consequences of RLS, but also constitute a rationale to prevent some, if not most, of these consequences.

Besides biological systems similarly altered in vitamin D deficiency and RLS, the role of vitamin D in the modulation of neurotransmitters reportedly altered in RLS, might indicate a rationale to consider D3 supplementation, at least as an add-on therapy, in resistant RLS with known vitamin D deficiency, once all other traditional RLS therapeutic options have been exploited. Above all, so far, scanty results from descriptive observational studies suggest that vitamin D supplementation should be encouraged along with iron therapy in most cases of augmentation to minimize opioid prescriptions and alleviate drug withdrawal.

Finally, evidence on the protective effects of vitamin D on developing dopamine neurons and pain pathways should encourage early supplementation trials with vitamin D in deficient pediatric populations at risk, such as children with ADHD or autism, and/or young women with strong genetic RLS predisposition, to mitigate or delay the occurrence of symptoms and their long-term consequences. Because of gender-related aspects in the prevalence and consequences of vitamin D deficiency, women rather than men should be the optimal target for preventative interventions.

Results from this review should also encourage, as a common practice, early life vitamin D and iron blood work in populations at risk for neurodevelopmental disorders, pain hypersensitivity and genetic predisposition to leg pain and motor symptoms. Longitudinal prospective studies should be designed to assess the preventative role of a correct D3 dosing in children and adolescent females with a genetic risk for idiopathic or comorbid RLS. Similarly, assessing and correcting D3 levels in conventionally treated long-term RLS should be evaluated against no supplementation in deficient subjects *vis-a-vis* neuro and cardiovascular RLS consequences and the risk of augmentation.

In addition to the future directions listed throughout the review, other directions are worth exploring. For example, animal models of RLS have targeted iron deficiency, the dopaminergic system [111,112,113] as well as the endogenous opiate system [83,84,85]. Vitamin D deficiency models have been created, but to our knowledge the clinical or biochemical particularities of RLS have not been investigated in these models [114,115]. A vitamin D deficiency model of RLS would be worth exploring to see if such a model is capable of mimicking the clinical and biochemical features of RLS.

In summary, this review documents the evidence for vitamin D deficiency and vitamin D supplementation in RLS. We also present data for the pathogenesis of RLS. In particular, we review the link between vitamin D and RLS to genetic predisposition, proteomic expression, cardiovascular disease, cerebrovascular disease, renal disease, inflammation, iron deficiency, and various neurotransmitters. Although the literature is not entirely consistent in affirming vitamin D deficiency in RLS or the amelioration of RLS symptoms with vitamin D therapy, the collective studies ocerall indicate that vitamin D deficiency is common enough in RLS patients to recommend that RLS patients should have their vitamin D levels checked and any deficiency corrected as a standard of care.
